# Neuroimmune signaling by extracellular vesicles

**DOI:** 10.18632/oncotarget.5249

**Published:** 2015-08-21

**Authors:** Stefan Momma

**Affiliations:** Institute of Neurology (Edinger Institute), Johann Wolfgang Goethe University Medical School, Frankfurt/Main, Germany

**Keywords:** Immunology and Microbiology Section, Immune response, Immunity

**Figure 1:**
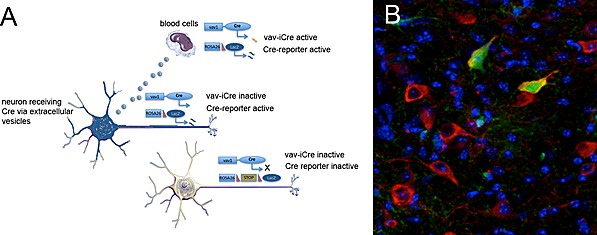
Transgenic reporter system to trace extracellular vesicle-mediated RNA transfer **A.** Functional scheme of a transgenic mouse where Cre recombinase expressed specifically in the hematopoietic lineage is crossed with a Cre reporter background. Cre is actively transcribed only in blood cells and not in neurons. When Cre mRNA is transferred to a neuron via EVs, Cre protein is generated and excises the stop signal, thereby irreversibly expressing the reporter gene. **B.** Reporter gene- (GFP, green), tyrosine hydroxylase-positive (red) dopaminergic neurons in the substantia nigra that received Cre mRNA via EVs from the blood after systemic inflammation. Image Credit: Sabrina Jung.

Systemic inflammation has a strong impact on brain function and contributes to the immediate and long-term development of various pathologies. The classic view is that a systemic inflammation influences the brain via the release of inflammatory cytokines (e.g. IL-1Δ, IL6, TNFα), which in turn activate microglia directly or via endothelial cells but may also act on neurons. As a neural mechanism, cytokines may activate the vagus nerve within the abdominal and thoracic cavity by relaying a signal to neuronal populations in the brainstem. Lately, other nonclassical ways of spreading disease from the periphery to the brain with a link to inflammation have been suggested, particularly for Parkinson's disease, but the nature of the responsible agent remains unclear. In the light of this latter concept, it is particularly interesting that we were recently able to identify the direct transfer of functional RNA from peripheral blood to neurons via extracellular vesicles [1]. Extracellular vesicles (EVs) are a heterogeneous population of small, membrane-bound particles containing multiple types of molecules such as lipids, ribonucleotides and proteins. It has been postulated that EVs are important regulators of the immune response [2], but most data are based on *in vitro* experiments or studies involving at least some *in vitro* manipulations, thereby questioning their physiological relevance. In contrast, for the first time we were able to demonstrate a transfer of functional RNA by EVs *in vivo*. This was achieved by using the Cre/LoxP system to visualize the contribution of blood cells to other organs, originally in the context of cell fusion (Figure 1) [3]. Briefly, expression of Cre recombinase specifically in blood cells leads to the release of Cre mRNA via EVs into the circulation that can then reach neurons in the brain, but only after peripheral inflammation. While we detect changes in the miRNA composition of EVs receiving neurons, currently we do not know whether this process is pathologic or acts as a rescue mechanism, trying to counteract cellular stress induced by yet other molecules. Also, the specificity of the response remains unclear, although our data suggest that the local permeability of the blood-brain barrier is a likely parameter.

In general, the visualization of RNA transfer by EVs *in vivo* will allow the identification of routes of communication between distant cells, which was previously not possible, and we could confirm the validity of our approach by giving the first account of tumor-to-host signaling [4]. Of particular interest in the light of our findings of a route from immune cells to the brain is the fact that the main target of malignant astrocytoma EVs appears to be immune cells infiltrating into the tumor mass. This could indicate the general possibility of a reverse information transfer from the brain to the immune system by EVs in a specific pathological context.

What distinguishes a communication between tissues on the basis of EVs from individual molecular entities such as cytokines? For one, increased complexity. An individual unit, i.e. a vesicle, can, in principle, simultaneously transfer a multitude of signaling molecules that induce an effect in their target cells by extracellular, or intracellular, interactions. Secondly, molecules that until now have not been considered as being transferred between cells such as intracellular proteins and RNAs may emerge as relevant mediators of pathology. As a consequence, EVs may serve as highly specific vehicles for the delivery of therapeutic molecules, particularly to the brain. Additionally, the very complexity of EVs may make them more suitable as a diagnostic tool rather than individual proteins that are more difficult to attribute to a specific disease. This latter approach of using EVs in diagnostics has recently been demonstrated for early pancreatic cancer [5] and using this type of diagnosis for neurodegenerative diseases is an intriguing possibility. However, we are just at the beginning of our understanding of the biology of EVs, let alone their physiological function *in vivo*. Clearly EVs are a heterogeneous collection of vesicles, but the biological significance of this heterogeneity is unknown [6]. Furthermore, whether RNAs contained in EVs are present in sufficient quantities to have a lasting impact on the receiving cell is a controversial issue. In summary, the novel concept of brain-blood interaction by extracellular vesicles in the context of inflammation has the potential to significantly advance our understanding of ways in which the immune system and the brain interact and contribute to neurodegenerative diseases and opens additional possibilities to be explored for new diagnostic strategies.
